# Association between Findings in Oral Health Screening and Body Mass Index: A Nation-Wide Longitudinal Study

**DOI:** 10.3390/ijerph182111062

**Published:** 2021-10-21

**Authors:** Yoonkyung Chang, Jimin Jeon, Jin-Woo Kim, Tae-Jin Song, Jinkwon Kim

**Affiliations:** 1 Department of Neurology, Mokdong Hospital, Ewha Womans University College of Medicine, Seoul 07985, Korea; tin1207@nate.com; 2 Department of Neurology, Yongin Severance Hospital, Yonsei University College of Medicine, Yongin-si 16995, Korea; jmk50040@gmail.com; 3 Department of Oral and Maxillofacial Surgery, School of Medicine, Ewha Womans University Medical Center, Mokdong Hospital, Seoul 07985, Korea; jinu600@gmail.com; 4 Department of Neurology, Seoul Hospital, Ewha Womans University College of Medicine, Seoul 07804, Korea

**Keywords:** body mass index, obesity, oral health, periodontitis, tooth brushing

## Abstract

Oral diseases, such as periodontitis and dental caries, can cause systemic inflammation as well as local effects, which is an important contributing factor for obesity. We aimed to investigate the change in body mass index (BMI) according to the presence of periodontitis and oral hygiene indicators. This study enrolled 110,068 participants from the national health screening cohort in Korea from 2009–2010 who underwent an oral health checkup. As oral hygiene indicators, the presence of periodontitis, dental caries, tooth loss, and tooth brushing were assessed. We constructed a linear mixed model for BMI. BMI was repeatedly measured in the health examination until 2015. In the multivariate linear mixed model for BMI, the presence of periodontitis (β = 0.0687, standard error (SE) = 0.0225, *p* = 0.002), dental caries (β = 0.0735, SE = 0.0152, *p* < 0.001), and tooth loss (β = 0.1328, SE = 0.0175, *p* < 0.001) were positively associated with BMI. In contrast, frequent tooth brushing (≥3 times/day) was negatively associated with BMI (β = −0.2610, SE = 0.0306, *p* < 0.001). The presence of periodontitis, dental caries, and tooth loss may be associated with higher BMI, whereas frequent tooth brushing may be related to lower BMI. Better oral hygiene might be associated with lower BMI. Further study is needed to determine the effect of oral health behavior and dental disease on obesity.

## 1. Introduction

Recently, the prevalence of obesity has steadily increased, becoming a worldwide phenomenon [1]. Furthermore, obesity is an independent and strong predictor of cardiovascular diseases, even without the interaction of other risk factors [2]. Thus, because obesity is a modifiable risk factor for cardiovascular disease, several strategies to control overweight and obesity have been considered, such as low fat or proper carbohydrate diets, physical activities, lifestyle modification, and use of medications for weight reduction [3]. However, the methods for controlling overweight and obesity are limited in clinical practice.

Periodontitis, dental caries, and tooth loss are common diseases reflecting poor oral conditions [4]. Periodontitis is defined as an inflammatory disease of the supporting tissues of teeth caused by specific microorganisms or groups of specific microorganisms, resulting in progressive destruction of the periodontal ligament and alveolar bone with periodontal pocket formation, gingival recession, or both [5]. Emerging evidence suggests that poor oral health and periodontitis can influence the initiation and/or progression of various diseases such as cancer, cardiovascular disease, metabolic disease, and degenerative diseases [6,7,8,9]. On the other hand, behaviors improving oral health may reduce the risk of medical illnesses [10,11]. One possible mechanism for this is that oral diseases not only induce local inflammatory effects that destroy the dentition and tooth-supporting tissues, but also cause systemic inflammation [4]. The enhanced inflammatory reaction is one of the main pathophysiologic mechanisms of obesity [12]. Dental caries are caused by a breakdown of the tooth enamel and extend to dentin and pulp tissues. This breakdown is the result of bacteria on teeth that break down foods and produce acid that destroys tooth microstructures and results in tooth decay. Dental caries or tooth loss may adversely affect nutrition intake, leading to malnutrition or obesity [13,14]. As complex chronic diseases, both periodontitis and dental caries have been indicated to share common risk factors that are inherited and acquired [15].

We hypothesized that poor oral health could contribute to the development and progression of obesity. Poor oral health can be indicated by the presence of periodontitis, dental caries, tooth loss, and infrequent tooth brushing behaviors. However, research on the relationship of oral health indicators with obesity is limited, especially in longitudinal settings. Therefore, we investigated whether the oral health indicators including periodontitis, dental caries, tooth loss, and tooth brushing behaviors have associations with longitudinal values in body mass index (BMI), using a nation-wide population-based health screening cohort in Korea.

## 2. Materials and Methods

### 2.1. Data Source

Our study utilized the National Health Insurance Service-National Health Screening Cohort (NHIS-HEALS) dataset in the Republic of Korea [16]. The Republic of Korea provides a public health insurance system with a single-payer organization of NHIS. Every two years, the NHIS provides a complimentary nation-wide health checkup program to all Korean aged ≥ 40 years. The NHIS-HEALS is a sample cohort of participants who participated in the free health screening program, which is constructed to offer relevant and useful health data for political and academic research [16]. The cohort dataset included serial health checkup data of consecutive participants, including their BMI, blood and urine test results, oral health checkup questionnaire, oral health evaluated by dentists, blood pressure measurements, and lifestyle survey results. In addition, NHIS-HEALS contains personal health claims data for the hospital visit, diagnosis (using the International Statistical Classification of Diseases and Related Health Problems 10th revision (ICD-10)), prescription, and procedures. The health claim resources were available until 31 December 2015, the loss of health insurance eligibility, or death. More detailed information of NHIS-HEALS is available in a prior publication [16].

### 2.2. Study Participants

**Figure 1:**
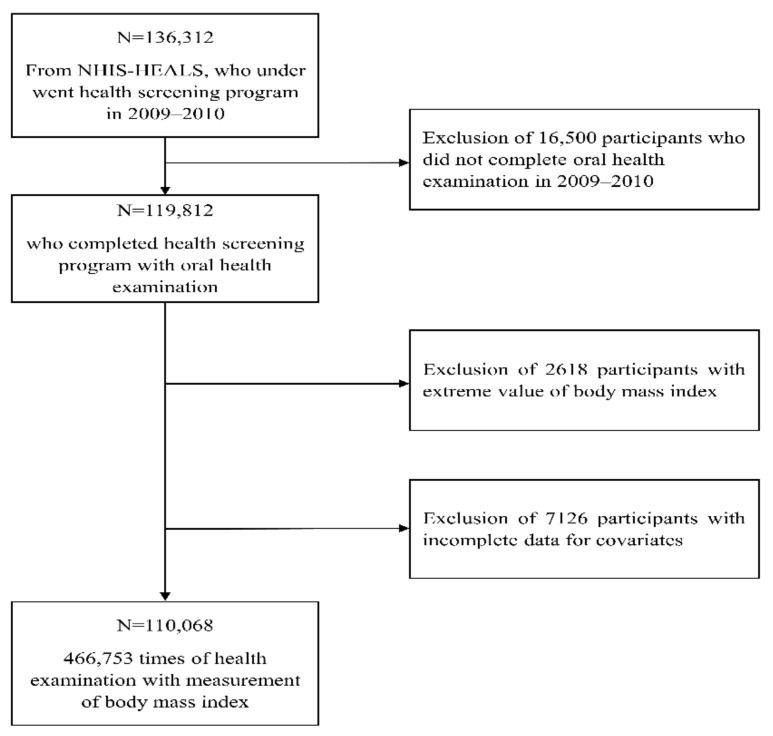
Flow chart showing the inclusion and exclusion criteria. NHIS–HEALS, National Health Insurance Service-National Health Screening Cohort in Korea.

This study enrolled participants from the NHIS-HEALS 2009–2010 dataset who had an oral health checkup as the baseline examination. We excluded participants with extreme BMI values (>32 kg/m^2^ or <17 kg/m^2^) and those who had missing data for at least one of the covariates in the NHIS-HEALS 2009–2010 dataset. A flow chart representing inclusion and exclusion criteria is shown in Figure 1. This study was approved by the Institutional Review Board of our institution (SEUMC 2020-08-18). The informed consent was waived due to the retrospective analysis based on the fully anonymized data.

### 2.3. Definition of Oral Health Indicators and Variables

The presence of dental caries and tooth loss were investigated by dentists at the baseline oral health checkup (2009–2010). Regarding dental caries, dentists investigated whether the participants had caries of permanent teeth, and finally, those who had at least one caries of permanent teeth were classified as having dental caries. Tooth loss was evaluated regardless of the cause, and we regarded the presence of a fixed dental prosthesis, implant with an abutment, and a third molar as toothlessness [15]. The presence of periodontitis was defined as if a diagnosis of acute periodontitis or chronic periodontitis (K052–053, respectively) was made more than twice by a dentist or if the participants underwent treatment for periodontal disease (U1010, U1020, U1051-1052, U1071-1072, and U1081-1083 claim codes) with K052 or K053 diagnosis for 1 year before the baseline oral health checkup. The K052 code included acute periodontitis/periodontal abscess (parodontal abscess) of gingival origin without sinus/acute apical periodontitis of pulpal origin/periapical abscess/periapical abscess with sinus/periodontal abscess (parodontal abscess) of gingival origin with sinus/acute apical periodontitis of pulpal origin/periapical abscess/periapical abscess with sinus/acute pericoronitis/other specified acute periodontitis/acute periodontitis, unspecified. The K053 code included chronic periodontitis/chronic simplex periodontitis/chronic complex periodontitis/chronic pericoronitis/other specified chronic periodontitis/chronic periodontitis, unspecified [10,11]. The frequency of daily tooth brushing was categorized as ‘0–1 time/day’, ‘2 times/day’, and ‘≥3 times/day’, based on a self-reported questionnaire.

We assessed data for sex, age, BMI, household income level (four quartiles), and presence of comorbidities (hypertension, diabetes mellitus, and chronic kidney disease) in the baseline health checkups. BMI was defined as one’s weight (kg) divided by the square of height (m^2^). BMI was serially measured in the health examination programs from the baseline health examination throughout the study period. Hypertension was defined when participants had health claims of blood pressure-lowering agents with ICD 10; I10–15, blood pressure ≥ 140/90 mmHg, or positive checking in a self-report questionnaire regarding hypertension. Diabetes mellitus was defined when participants had health claims of anti-diabetic drugs with diagnostic codes with ICD 10; E08–11, E13–14, fasting blood glucose >7.0 mmol/L, or positive checking in a self-report questionnaire for diabetes mellitus. Chronic kidney disease was determined by diagnostic codes of ICD 10; N18.1–N18.5 and N18.9, or an estimated glomerular filtration rate <60 mL/min/1.73 m^2^ [6]. From the repeated health checkups conducted during the follow-up, data for smoking status (current, former, never), alcohol consumption (frequency per week), physical activity (days per week), and laboratory findings (aspartate aminotransferase, alanine aminotransferase, and total cholesterol) were longitudinally collected [8,11,17].

### 2.4. Statistical Analysis

The comparison for categorical variables and continuous variables between the two groups were assessed using chi-square and independent t-tests, respectively. Differences between groups according to the frequency of daily tooth brushing were investigated using the chi-square test for trend or Spearman correlation analysis. To evaluate the variables related to BMI, we developed a linear mixed model with variables of fixed effects (oral health indicators, sex, age, household income, presence of comorbidities, smoking status, alcohol consumption, physical activity, aspartate aminotransferase, alanine aminotransferase, and total cholesterol). Statistical analyses were executed using R software, version 3.3.3 (R Foundation for Statistical Computing, Vienna, Austria), and SAS 9.4 version (SAS Inc., Cary, NC, USA). Two-sided P-values less than 0.05 were considered significant.

## 3. Results

### 3.1. Demographics of the Study Participants

Based on inclusion and exclusion criteria, 110,068 participants of a health screening program with oral health checkups in 2009–2010 were included (Figure 1). A comparison of the characteristics of participants who did and did not undergo complete oral examination in the NHIS-HEALS is shown in Table S1. Participants who underwent oral health examinations were predominantly male, younger in age, and had a higher household income as compared to those who did not. The prevalence of diabetes mellitus, hypertension, and chronic kidney disease was lower in participants who received oral health examinations. Finally, 466,753 BMI measurements were included from the repeated health examinations conducted for the included participants. Table 1 shows the serial measurements of BMI levels. The mean baseline BMI value was 23.95 (2.66) kg/m^2^. The median number of BMI measurements per participant was 4 (interquartile range (IQR), 3–6), and the median follow-up time was 5.14 years (IQR, 4.06–5.95 years).

The baseline characteristics of the study participants are shown in Table 2. The mean age was 56.6 (7.8) years at the baseline examination, and the proportion of males was 60.0%. The prevalence of periodontitis, dental caries, and tooth loss was 13.1%, 51.1%, and 25.8%, respectively. Moreover, 49.2% of participants presented a tooth brushing frequency of ≥3 times/day (Table 2). Characteristics of participants according to the presence of periodontitis and frequency of tooth brushing are shown in Table 3 and Table S2. Participants with periodontitis were predominantly male and older, presenting the highest quartile of household income, a higher proportion of current smokers, and a higher frequency of alcohol consumption and physical activity as compared to those without periodontitis. The BMIs of participants with periodontitis were significantly higher than those of participants without periodontitis. In addition, participants with periodontitis were more commonly accompanied by comorbidities such as hypertension, diabetes mellitus, and chronic kidney disease.

### 3.2. Association of Oral Health Indicators with BMI

**Figure 2:**
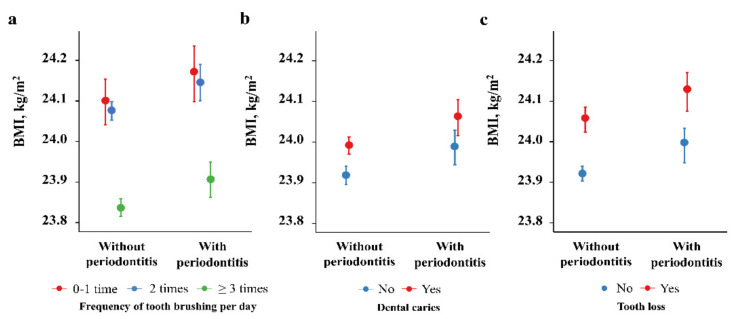
Body mass index according to the presence of periodontitis and oral health indicators: (**a**) BMI of patients according to frequency of tooth brushing, (**b**) BMI of patients according to the presence of dental caries, (**c**) BMI of patients according to the presence of tooth loss. Data are estimated as mean and 95% confidence intervals based on the multivariable linear mixed models for body mass index (fixed effect variables are the same as the models in Table 4). There was no significant interaction effect between periodontitis and other oral health indicators on body mass index (*p* value for interaction >0.05).

A multivariate linear mixed model was used to evaluate the longitudinal changes in BMI (Table 4). There was a positive relationship between presence of periodontitis and BMI (β = 0.0687, standard error = 0.0225, *p* = 0.002). Furthermore, the presence of dental caries and tooth loss were both positively related with BMI (β = 0.0735, standard error = 0.0152, *p* < 0.001; and β = 0.1328, standard error = 0.0175, *p* < 0.001, respectively). In contrast, there was a significant negative correlation between tooth brushing frequency ≥3 times/day and BMI (β = −0.2610, standard error = 0.0306, *p* < 0.001). These results mean that persons with periodontitis, dental caries, and tooth loss (positive value of β for BMI) had higher values of BMI than those without them. On the other hand, persons with frequent tooth brushing had lower values of BMI (negative value of β for BMI). In the linear mixed model for BMI, we did not find a significant interaction between time and oral health indicators (dental caries, tooth loss, periodontitis, and frequency of tooth brushing). When we evaluated the interaction effect between periodontitis and other oral health indicators on BMI in the model, no significant interaction was noted (Figure 2, all *p* value for interaction > 0.05). These data suggested that the significant association between the oral health indicators and body mass index were consistent regardless of the presence of periodontitis.

## 4. Discussion

Our study demonstrated that poor oral health-related diseases or indicators, such as periodontitis, dental caries, and tooth loss were positively associated with longitudinal changes in BMI, suggesting an increased risk of obesity. Moreover, improved oral health behavior of frequent tooth brushing was negatively associated with BMI.

Previous studies have evaluated the association between periodontitis, poor oral health, and obesity. In a meta-analysis of previous cross-sectional studies, periodontitis was strongly associated with increased BMI [18]. Moreover, the severity of periodontitis positively correlated with the degree of obesity [19]. Furthermore, in previous studies, periodontitis was closely associated with oral hygiene indicators [4,5]. In our study, with respect to the association of periodontitis with BMI, there was no statistical interaction between periodontitis and other oral hygiene indicators. Therefore, our findings are meaningful in that our results showed that periodontitis was independently associated with BMI regardless of other oral hygiene indicators.

Increased tooth loss, a marker of poor oral health, was also significantly associated with obesity [20]. In a study on older people in Brazil, tooth loss was positively associated with obesity [21]. However, the relationship between obesity and oral health remains inconclusive, especially in longitudinal settings. Our study is in line with these previous studies, and it is relevant because it provides evidence regarding the association of periodontitis and poor oral health indicators with the longitudinal changes in BMI.

In our study, the general population presenting frequent tooth brushing habits had a low BMI, even after adjusting for the confounding factors of oral health indicators. In a previous cross-sectional study, less-frequent tooth brushing was associated obesity, even after adjusting for oral health indicators (odds ratio: 1.22 and 1.48 for tooth brushing frequency of 1 time/day and 0 time/day, respectively) [22]. In addition, a recent general population-based longitudinal study of 4537 participants in Japan demonstrated that a low frequency of tooth brushing (≤1 time/day) was associated with the occurrence of obesity (prevalence rate ratio: 1.77, 95% confidence interval: 1.12–2.80) [23]. Our research results were similar to these previous studies; particularly, this longitudinal cohort study suggested a negative association between frequent tooth brushing and BMI.

Inflammation is thought to be an important factor that can explain the association between poor oral health indicators and obesity [24]. In the presence of poor oral health, including periodontitis, an inflammatory reaction occurs in the oral cavity [25]. The enhanced local inflammation can spread systemically through tooth loss or periodontal pockets, leading to increased secretion of endotoxins or inflammatory cytokines [26,27]. This systemic inflammation plays a decisive role in the pathogenesis and development of obesity [28]. The link between tooth brushing and obesity can be explained by a leptin-related pathway that controls the balance of appetite and energy. Intraoral proprioception-related pathways are linked to histamine-hypothalamic pathways, which are closely related to leptin signaling pathways [29]. Therefore, it is presumed that stimulation of the oral cavity and possible maintaining oral health by frequent tooth brushing can suppress appetite and reduce the risk of obesity. Further research in elucidating the mechanism of oral diseases including periodontitis, dental caries, and tooth loss should be followed.

Our study had some limitations. First, our dataset included only Asian people, mainly Koreans. Second, our dataset may have recall bias because our tooth brushing dataset was collected using a self-reported questionnaire. Third, the burden and location of dental caries, tooth loss, and periodontitis were not investigated due to the lack of detailed data in the nation-wide oral health checkup program used. Fourth, for our dataset, our study was conducted using the 2009–2010 health screening cohort dataset. At this time, because there was a lack of consensus on periodontitis in the NHIS-HEALS, probing was not performed on all participants, and it was not possible to confirm the agreements regarding the presence or severity of periodontitis. For this reason, we defined periodontitis with the ICD-10 codes and actual treatment claim codes in our study. Further research using a clear definition of periodontitis is needed in the future. Fifth, information regarding education levels, marital status, and other biomarkers representing inflammatory reactions were not available in the NHIS-HEALS dataset. Sixth, our observational study was without any interventions, and it cannot clearly suggest the mechanisms that explain the relationship of periodontitis and oral health indicators or behaviors with longitudinal changes in BMI. Seventh, our dataset does not provide information on the causes of tooth loss or types of dental caries, that is, active and treated arrested caries. Eighth, because the design of our study was a retrospective observational study, we checked the baseline characteristics of oral health from the participants only once, and the oral health behaviors and comorbidities may have changed over time.

## 5. Conclusions

In conclusion, the presence of periodontitis, dental caries, and tooth loss may be associated with a higher BMI, whereas frequent tooth brushing may be related to lower BMI. Better oral health might be associated with lower BMI. Further study is needed to determine the effects of oral health behavior and dental disease on obesity.

## Figures and Tables

**Table 1:** Serial measurement of body mass index.

Time of BMI Measurement Since Baseline	Baseline (2009–2010)	0–1 Year	1–2 Years	2–3 Years	3–4 Years	4–5 Years	5–6 Years	Over 6 Years
Mean (SD), BMI	23.95 (2.66)	23.90 (2.60)	23.93 (2.63)	23.95 (2.63)	23.93 (2.64)	23.96 (2.66)	24.01 (2.64)	24.08 (2.67)
Number of BMI measures	110,068	24,606	68,776	65,869	63,392	63,820	47,354	22,868

BMI, body mass index; SD, standard deviation.

**Table 2:** Characteristics of patients at baseline examination.

Variable	Values
Number of patients	110,068
Sex, male	66,040 (60.0)
Age, year	56.6 (7.8)
Household income	
Q1, lowest	26,322 (23.9)
Q2	25,573 (23.2)
Q3	30,966 (28.1)
Q4, highest	27,207 (24.7)
Smoking status	
Never	56,600 (51.4)
Former	32,994 (30.0)
Current	20,474 (18.6)
Alcohol consumption, frequency per week	
<1 time	60,521 (55.0)
1–2 times	34,166 (31.0)
3–4 times	10,856 (9.9)
≥5 times	4525 (4.1)
Physical activity, days per week	
<1 day	34,040 (30.9)
1–4 days	42,313 (38.4)
≥5 days	33,715 (30.6)
Anthropometric measurements	
Systolic blood pressure, mmHg	124.1 (14.6)
Diastolic blood pressure, mmHg	77.2 (9.8)
Body mass index, kg/m^2^	23.95 (2.66)
Comorbidities	
Hypertension	45,131 (41.0)
Diabetes mellitus	15,287 (13.9)
Chronic kidney disease	11,752 (10.7)
Laboratory findings	
Fasting glucose, mmol/L	5.58 (1.33)
Aspartate aminotransferase, U/L	26.0 (16.0)
Alanine aminotransferase, U/L	25.1 (18.7)
Total cholesterol	5.0 (0.8)
Oral health status	
Presence of periodontitis	14,470 (13.1)
Presence of dental caries	56,205 (51.1)
Presence of tooth loss	28,402 (25.8)
Oral health care	
Frequency of tooth brushing per day	
0–1 time	7953 (7.2)
2 times	47,996 (43.6)
≥3 times	54,119 (49.2)

Data are expressed as mean (standard deviation) or n (%). Q: quartile.

**Table 3:** Characteristics of patients according to the presence of periodontitis.

Variable	Without Periodontitis N = 95,598	With Periodontitis N = 14,470	*p* Value
Sex, male	56,520 (59.1)	9520 (65.8)	<0.001
Age, year	56.5 (7.8)	57.3 (7.8)	<0.001
Household income			<0.001
Q1, lowest	23,099 (24.2)	3223 (22.3)	
Q2	22,427 (23.5)	3146 (21.7)	
Q3	26,845 (28.1)	4121 (28.5)	
Q4, highest	23,227 (24.3)	3980 (27.5)	
Smoking status			<0.001
Never	50,011 (52.3)	6589 (45.5)	
Former	28,158 (29.5)	4836 (33.4)	
Current	17,429 (18.2)	3045 (21.0)	
Alcohol consumption, frequency per week			<0.001
<1 time	52,878 (55.3)	7643 (52.8)	
1–2 times	29,490 (30.8)	4676 (32.3)	
3–4 times	9330 (9.8)	1526 (10.5)	
≥5 times	3900 (4.1)	625 (4.3)	
Physical activity, days per week			<0.001
<1 day	29,877 (31.3)	4163 (28.8)	
1–4 days	36,707 (38.4)	5606 (38.7)	
≥5 days	29,014 (30.4)	4701 (32.5)	
Anthropometric measurements			
Systolic blood pressure, mmHg	124.1 (14.7)	124.2 (14.4)	0.683
Diastolic blood pressure, mmHg	77.2 (9.8)	77.2 (9.7)	0.549
Body mass index, kg/m^2^	23.9 (2.7)	24.0 (2.7)	<0.001
Comorbidities			
Hypertension	38,877 (40.7)	6254 (43.2)	<0.001
Diabetes mellitus	12,876 (13.5)	2411 (16.7)	<0.001
Chronic kidney disease	9981 (10.4)	1771 (12.2)	<0.001
Laboratory findings			
Fasting glucose, mmol/L	5.56 (1.3)	5.7 (1.5)	<0.001
Aspartate aminotransferase, U/L	26.0 (16.2)	26.1 (14.4)	0.175
Alanine aminotransferase, U/L	25.0 (18.7)	25.59 (18.4)	<0.001
Total cholesterol	5.0 (0.4)	5.0 (1.1)	0.089
Oral health status			
Presence of dental caries	50,287 (52.6)	5918 (40.9)	<0.001
Presence of tooth loss	23,695 (24.8)	4707 (32.5)	<0.001
Oral health care			
Frequency of tooth brushing per day			<0.001
0–1 time	7068 (7.4)	885 (6.1)	
2 times	42,115 (44.1)	5881 (40.6)	
≥3 times	46,415 (48.6)	7704 (53.2)	

Data are expressed as mean (standard deviation) or n (%). *p* value is derived from the independent t-test and chi-square test. Q: quartile.

**Table 4:** Association of periodontitis and oral health indicators with body mass index.

Variables	Body Mass Index, kg/m^2^		
	Beta	Standard Error	*p* Value
Oral health status			
Presence of periodontitis	0.0687	0.0225	0.002
Presence of dental caries	0.0735	0.0152	<0.001
Presence of tooth loss	0.1328	0.0175	<0.001
Oral health care			
Frequency of tooth brushing per day			
0–1 time	Ref		
2 times	−0.0216	0.0306	0.481
≥3 times	−0.2610	0.0306	<0.001

Data are derived from a multivariable linear mixed model for longitudinal data of body mass index, which includes sex, age, household income, smoking status, alcohol consumption, physical activity, the presence of diabetes mellitus, hypertension, and chronic kidney disease, aspartate aminotransferase, alanine aminotransferase, and total cholesterol level as fixed effects.

## Data Availability

The data that support the findings of this study are available from NHIS-HEALS, but restrictions apply to the availability of these data, which were used under license for the current study and so are not publicly available.

## Supplementary Materials

The following are available online at https://www.mdpi.com/article/10.3390/ijerph182111062/s1, Table S1: Comparison between the characteristics of patients who completed oral health examination and those who did not, Table S2: Characteristics of the study participants according to the frequency of tooth brushing per day.
