# Supplementary material for: Association between Findings in Oral Health Screening and Body Mass Index: A Nation-Wide Longitudinal Study
Source: Int J Environ Res Public Health. 2021 Oct 21;18(21):11062. doi: 10.3390/ijerph182111062 (PMC8582665; doi:10.3390/ijerph182111062)
Supplement: Supplementary file 1 [file ijerph-18-11062-s001.zip › Table S1.pdf]

Table S1. Comparison between the characteristics of patients who completed oral health examination and those who did not.

**Variables**	**With oral health examination (N=119812)**	**Without oral health examination (excluded from the study, N=16500)**	**Standardized difference**	***p* value**
Sex, male	71699 (59.8)	9480 (57.5)	0.049	<0.001
Age, year	56.62 (7.84)	60.82 (7.94)	0.532	<0.001
Household income			0.136	<0.001
Q1, lowest	28756 (24.0)	4516 (27.4)		
Q2,	27887 (23.3)	4200 (25.5)		
Q3,	33514 (28.0)	4551 (27.6)		
Q4, highest	29655 (24.8)	3233 (19.6)		
Smoking status *			0.048	<0.001
Never	57339 (51.5)	8054 (53.5)		
Former	33246 (29.9)	4184 (27.8)		
Current	20737 (18.6)	2829 (18.8)		
Alcohol consumption, frequency per week *			0.151	<0.001
<1 time	61295 (55.1)	9081 (60.3)		
1–2 times	34478 (31.0)	3767 (25.0)		
3-4 times	11000 (9.9)	1379 (9.2)		
≥5 times	4549 (4.1)	840 (5.6)		
Physical activity, days per week *			0.111	<0.001
<1 day	34514 (31.0)	5434 (36.1)		
1–4 days	42822 (38.5)	5198 (34.5)		
≥5 days	33986 (30.5)	4435 (29.4)		
Comorbidities *				
Hypertension	48764 (40.7)	8051 (48.8)	0.163	<0.001
Diabetes mellitus	16393 (13.7)	2915 (17.7)	0.114	<0.001
Chronic kidney disease	11098 (10.0)	1655 (11.0)	0.033	<0.001

Data are expressed as mean (standard deviation) or n (%). *p* value is derived from the independent t-test and chi-square test. Q: quartile. * There were 9923 participants for whom values of smoking status, alcohol consumption, physical activity, and comorbidities were missing.
