# Supplementary material for: Association between Findings in Oral Health Screening and Body Mass Index: A Nation-Wide Longitudinal Study
Source: Int J Environ Res Public Health. 2021 Oct 21;18(21):11062. doi: 10.3390/ijerph182111062 (PMC8582665; doi:10.3390/ijerph182111062)
Supplement: Supplementary file 1 [file ijerph-18-11062-s001.zip › Table S2.pdf]

Table S2. Characteristics of the study participants according to the frequency of tooth brushing per day.

Variables	Frequency of tooth brushing per day			p value
	0-1<br>N=7953	2<br>N=47996	≥3<br>N=54119	
Sex, male	5890 (74.1)	27462 (57.2)	32688 (60.4)	<0.001
Age, year	60.13 (8.88)	57.21 (7.88)	55.47 (7.27)	<0.001
Household income				<0.001
Q1, lowest	2161 (27.2)	12259 (25.5)	11902 (22.0)	
Q2,	2105 (26.5)	12434 (25.9)	11034 (20.4)	
Q3,	2202 (27.7)	13222 (27.5)	15542 (28.7)	
Q4, highest	1458 (18.7)	10081 (21.0)	15641 (28.9)	
Smoking status				<0.001
Never	3175 (39.9)	24980 (52.0)	28445 (52.6)	
Former	2776 (34.9)	13463 (28.1)	16755 (31.0)	
Current	2002 (25.2)	9553 (19.9)	8919 (16.5)	
Alcohol consumption, frequency per week				<0.001
<1 time	3987 (50.1)	27043 (56.3)	29491 (54.5)	
1–2 times	2285 (28.7)	13827 (28.8)	18054 (33.4)	
3-4 times	945 (11.9)	4976 (10.4)	4935 (9.1)	
≥5 times	736 (9.3)	2150 (4.5)	1639 (3.0)	
Physical activity, days per week				<0.001
<1 day	3315 (41.7)	16290 (33.9)	14435 (26.7)	

1–4 days	2669 (33.6)	17950 (37.4)	21694 (40.1)	
≥5 days	1969 (24.8)	13756 (28.7)	17990 (33.2)	
Anthropometric measurements				
Systolic blood pressure, mmHg	125.83 (15.22)	124.54 (14.73)	123.54 (14.42)	<0.001
Diastolic blood pressure, mmHg	77.74 (9.86)	77.27 (9.80)	77.12 (9.73)	<0.001
Body mass index, kg/m^2	24.16 (2.81)	24.06 (2.68)	23.82 (2.60)	<0.001
Comorbidities				
Hypertension	3980 (50.0)	20471 (42.7)	20680 (38.2)	<0.001
Diabetes mellitus	1527 (19.2)	6931 (14.4)	6829 (12.6)	<0.001
Chronic kidney disease	960 (12.1)	5244 (10.9)	5548 (10.3)	0.001
Laboratory findings				
Fasting glucose, mmol/L	5.78 (1.60)	5.61 (1.34)	5.52 (1.27)	<0.001
Aspartate aminotransferase, U/L	27.23 (18.13)	26.14 (16.68)	25.62(14.87)	<0.001
Alanine aminotransferase, U/L	26.19 (19.38)	25.32 (19.95)	24.67 (17.41)	<0.001
Oral health status				
Presence of periodontitis	885 (11.1)	5881 (12.3)	7704 (14.2)	<0.001
Presence of dental caries	4375 (55.0)	25695 (53.5)	26135 (48.3)	<0.001
Presence of tooth loss	2520 (31.7)	13153 (27.4)	12729 (23.5)	<0.001

Data are expressed as mean (standard deviation) or n (%). *p* value is derived from the chi-square for trend and Spearman correlation test. Q: quartile.
